# Long-Term Cardiovascular Fitness Is Associated with Auditory Attentional Control in Old Adults: Neuro-Behavioral Evidence

**DOI:** 10.1371/journal.pone.0074539

**Published:** 2013-09-04

**Authors:** Stephan Getzmann, Michael Falkenstein, Patrick D. Gajewski

**Affiliations:** Leibniz Research Centre for Working Environment and Human Factors at the Technical University of Dortmund (IfADo), Dortmund, Germany; UNLV, United States of America

## Abstract

It has been shown that healthy aging affects the ability to focus attention on a given task and to ignore distractors. Here, we asked whether long-term physical activity is associated with lower susceptibility to distraction of auditory attention, and how physically active and inactive seniors may differ regarding subcomponents of auditory attention. An auditory duration discrimination task was employed, and involuntary attentional shifts to task-irrelevant rare frequency deviations and subsequent reorientation were studied by analysis of behavioral data and event-related potential measures. The frequency deviations impaired performance more in physically inactive than active seniors. This was accompanied by a stronger frontal positivity (P3a) and increased activation of anterior cingulate cortex, suggesting a stronger involuntary shift of attention towards task-irrelevant stimulus features in inactive compared to active seniors. These results indicate a positive relationship between physical fitness and attentional control in elderly, presumably due to more focused attentional resources and enhanced inhibition of irrelevant stimulus features.

## Introduction

Healthy aging is associated with declines in various cognitive functions such as working memory capacity, processing speed, and attentional and inhibitory control [1]. As a consequence, goal-directed behavior of older adults often suffers from deficits in inhibiting irrelevant stimuli [2]. Deficits usually become manifest in complex task settings, in which concurring stimuli are present, and in which top-down attentional control is needed to focus attention on a relevant and to ignore irrelevant events. Such deficits are not inevitable, and there is increasing evidence that physical fitness can help to counteract age-related declines in cognitive performance. Consistently, reviews and meta-analyses suggested that aerobic exercise yields not only improvement in physical fitness and mood, but also in cognition across an array of populations, including elderly [3], [4]. The impact of aerobic exercise on cognition yields a moderate effect with the strongest and most consistent benefit in executive functions [4], [5].

Here, we asked whether physical fitness is associated with the interplay of distraction and orientation-related attentional processes in seniors. An auditory distraction paradigm was hereto employed that has been proven to be well suited to examine age-related declines in cognitive sub-processes underlying attentional and inhibitory control [6]–[9]. A sequence of repeated tones was intermixed with occasional irregular tones violating the repetition, and subjects had to respond to the *standard* tones, while ignoring *deviant* tone features [10]. The specific requirements of this task have been described within a three-stage model of distraction [11], [12]: The first stage of *regularity extraction* and *deviance-detection* comprises the filtering of task-relevant information out of a stream of ongoing stimulation, and the automatic detection of task-irrelevant information. At the second stage of *involuntary attention-switching* the deviant information may lead to involuntary attention shifts which are – in a final stage of *reorientation* – compensated for by mechanisms restoring the optimal attention-set relevant for a given task.

The basic cognitive sub-processes of this distraction-orientation-refocusing cycle can be distinguished by analysis of the event-related potentials (ERPs): In contrast to the standard tones, deviant stimuli typically evoke the fronto-central mismatch negativity (MMN), a physiological correlate of pre-attentive deviance detection [13], [14]. The MMN is usually followed by the fronto-central P3a [15], a correlate of an involuntary attention-switching mechanism [12], [15], [16]. Finally, the late fronto-central reorienting negativity (RON) is assumed to reflect re-allocation of attention to the relevant task after distraction by the deviant features [10], [11]. Both standard and deviant stimuli usually produce a fronto-central N1-P2 complex that is followed by the parietal P3b. The N1-P2 complex is assumed to reflect the automatic detection of auditory sensory input and influences of early attention and orientation processes (e.g., [17], [18]), while the P3b has been related to the allocation of working memory and task-relevant processing resources [19].

Previous studies have shown that the interplay of deviance detection, involuntary attention shifts and top-down attentional control is sensitive towards age-related processes [6]–[8], [20]–[22], indicating an increased susceptibility to distracting stimuli in elderly. In particular, an age-related reduction of MMN relative to younger adults has been reported suggesting specific deficits in encoding or retention of sensory information [20], [21], [23]. Also, age-related changes in P3a [6], [7], [22], [24] and RON [6], [7], [22] suggest attentional orienting and reorienting to contribute to deficits observed in elderly.

In the present study, physically active and inactive seniors discriminated the duration of short and long tones that were either of high-probability standard frequency or of low-probability deviant frequencies [10], [11]. Thus, the participants had to concentrate on the task-relevant tone feature (i.e., its duration), while ignoring the distracting task-irrelevant tone feature (i.e., its pitch). Assuming a close relationship between physical fitness and cognitive sub-processes underlying the distraction-orientation-refocusing cycle described above, active participants were expected to show a better performance, i.e. less distraction by the deviant tones, than their inactive counterparts. In order to reveal potential sources of performance differences between the active and inactive group, ERPs on standard and deviant stimuli were analyzed: Significant differences in N1 and MMN would suggest more severe deficits in the inactive group in sensory encoding and deviance detection, respectively, while differences in P3a and RON would suggest deficits in attentional orienting and reorienting. Taken together, the study investigated whether physical fitness might counteract auditory distraction in elderly and, if so, at what level cognitive sub-processes benefit most from physical fitness. We combined electroencephalography with simultaneous behavioral measures to investigate neural correlates of the distraction-orientation-refocusing cycle in elderly, thus addressing the sources of the potential benefit of long-term cardiovascular fitness on age-related deficits in attentional control.

## Materials and Methods

### Ethics Statement

This study conformed to the Code of Ethics of the World Medical Association (Declaration of Helsinki), printed in the British Medical Journal (18 July 1964). All patients gave their written informed consent to participate in this study, which was specifically approved by the local Ethical Committee of the Leibniz association (Ethics Commission of the Research Society for Occupational Physiology and Safety; Chair: Prof. Dr. Seeber).

### Participants

A group of 32 healthy male volunteers took part in the study, consisting of 16 physically active (mean age 72.6 years; range 66–79 years) and 16 inactive seniors (mean age 72.6 years; range 66–87 years). The participants of the active group were recruited from a local sports club. The data of the inactive group were gathered as part of a training study in which a total of 129 elderly adults (86 female; mean age 70.2 years; range 63–88 years) took part. These participants were recruited through a number of newspaper advertisements and flyers distributed in the city of Dortmund (Germany). The training study comprised a pre- and a post-measurement [25], with only the pre-measurement data being reported here. In order to constitute two groups consisting of athletic (physically active) and non-athletic (physically inactive) participants, 16 male adults were selected from the training study who matched with the physically active participants regarding age and education (see below) in almost the same manner. All participants met the following criteria: They were at least 65 years, mentally fit, living independently and self-paced, and having sufficient or corrected visual and auditory acuity. Exclusion criterions were history of cardio-vascular, psychiatric, neurological, motor or oncologic diseases, and psychopharmacologic or hormonal therapy. The scope of the study was explained to the participants and they gave written informed consent before any study protocol was commenced.

### Neuropsychological and Physical Testing

All participants underwent neuropsychological and physical assessment. In the physical test session the participants’ physical efficiency [26] (Physical Work Capacity PWC_130_) was measured on a bicycle ergometer. The aim of this test was to predict the absolute power output at a projected heart rate of 130 beats per minute. In addition to the maximum amount of power, the power-to-weight ratio (i.e., the power that a person is able to generate divided by his or her body weight) was computed as a measure of relative physical performance, reflecting the ability to generate the aerobic power in the most efficient manner. There were highly significant differences regarding physical fitness between the active and inactive group, in both conditions: maximum amount of power and power-to-weight ratio (Tab. 1). In addition to the physical assessment, an adapted version of the Lüdenscheider Activity Questionnaire (Lüdenscheider Aktivitätsfragebogen. Höltke and Jakob, 2002**,** Sportmedizin Hellersen, Lüdenscheid, Germany) was employed in order to measure the amount of physical activity of the participants. This questionnaire consisted of 13 items that survey daily motor activity in professional and private life (for example, how much time a person spent walking, swimming, bicycling, jogging, or gardening during the last week). The individual items were combined into a single activity index, ranging from 1.0 (low) to 4.0 (high). The results of the activity questionnaire were in line with the physical assessment, with significant correlations of activity level and maximum amount of power (*r* = 0.55; *p*<0.005), and power-to-weight ratio (*r* = 0.63; *p*<0.001). Moreover, the activity questionnaire indicated a significantly higher level of physical activity in the active than inactive group (Tab. 1). In contrast, the two groups did not differ in age, weight, height, level of education, or in general cognitive status (assessed by the Mini Mental State Examination [27], MMSE). In sum, the active and inactive groups were comparable in all sociodemographic and neuropsychological variables, but clearly differed in activity level and physical fitness.

**Table 1:** Sample characteristics and results of the sociodemographic, neuropsychological, and physical assessment for the active and inactive groups.

		Active	Inactive	*F* (1,30)	*p*
Number		n = 16	n = 16		
Age [years]		73.0 (5.1)	73.2 (4.4)	0.01	ns.
Weight [kg]		79.8 (8.8)	84.1 (11.2)	1.49	ns.
Height [cm]		175.3	175.7	0.06	ns.
BMI		25.9 (0.7)	27.2 (0.7)	1.55	ns.
Activity level		3.2 (0.7)	1.8 (0.6)	43.36	<0.001
Ergometric data	peak work [W]	137 (19)	111 (21)	13.20	<0.01
	watts per weight	1.75 (0.30)	1.34 (0.29)	15.93	<0.001
Mean Level of Education		4.2 (0.6)	3.8 (1.2)	0.83	ns.
MMSE		29.3 (0.8)	28.8 (0.9)	2.14	ns.

Significance level was set at *p*<0.05. Key: Activity level: (1) low, (4) high; BMI, Body Mass Index; Mean Level of Education: No degree (1), Primary (2), Secondary general (3), Intermediate secondary (4), Gymnasium (5); MMSE, Mini Mental State Examination.

### Stimuli, Task, and Procedure

Stimuli, task, and procedure have been described in detail in a related study [9]. The auditory stimuli were sine waves composed of base frequencies of either 500 Hz, 1000 Hz, or 2000 Hz. 80% of the stimuli were frequent standard stimuli (1000 Hz), and 20% were rare deviant stimuli (either 500 Hz or 2000 Hz, each 10%). The sequence of standard and deviant stimuli was pseudo-randomized. 50% of the tones were short (200 ms), and 50% were long (400 ms) (both including 5 ms rise and 5 ms fall times), presented with equal probability. The sound stimuli were presented binaurally using stereo headphones (AKG, K271 Studio) at the intensity of 70 dB(A). During testing, the participants sat on a comfortable chair in a dimly lit and quite room. They had to discriminate the duration of the tones using a two-alternative forced-choice duration discrimination task. They had to press one response button for short and another for long tones irrespective of the tone pitch. The response buttons were held in the subject’s hands. The duration-hand contingency was counterbalanced between participants. Participants were instructed to respond in a fast but accurate manner. To avoid EEG alpha-activity and wandering eye-movements during the recordings, participants were instructed to keep their eyes open and to focus on a visual fixation point presented on a monitor placed in front of them. The participants were given a written instruction that explained the task, and the instruction was also explained by the experimenter before starting the test. No feedback was given to the participants at any time during the experiment.

At the beginning of the session, the participants carried out a short training until the task was familiar. To ensure that the participants were able to reliably distinguish the tone pitches of the frequent and deviant stimuli, samples of the tones were presented, and the participants were asked whether they can hear the sounds, and whether they perceive the differences in pitch. All participants performed this task without any problems. Then, the participants completed two test blocks interrupted by a rest break. A test block consisted of 120 trials (48 short and 48 long standard tones and 12 short and 12 long deviant tones). The stimulus onset asynchrony was 1400 ms. The timing of the stimuli and the recording of the participants responses were controlled by custom-written software. Response times (RTs) were measured by a high-resolution timer interface connected with the external response buttons.

### Data Recording

The continuous EEG (amplifier bandpass 0.01–140 Hz) was sampled at 2048 Hz using 32 Ag/AgCl electrodes mounted on an elastic cap according to the extended 10–20 system. The montage included 8 midline sites and 12 sites on each hemisphere. Horizontal and vertical eye positions were recorded by EOG using 4 electrodes positioned around both eyes. The ground electrode was placed on the center of the forehead, just above the nasion. Two additional electrodes were placed on the left and right mastoids (M1 and M2). Electrode impedance was kept below 10 kΩ. The raw data were offline downscaled to a sampling rate of 1000 Hz, band-pass filtered (cut-off frequencies 0.05 and 17 Hz), re-referenced to linked mastoids, and segmented into 1300-ms stimulus-locked epochs covering the period from −100 to 1200 ms relative to tone onset, using the Brain Vision Analyzer software (Version 1.05; Brain Products, Munich, Germany). The data were corrected for ocular artifacts using the Gratton and Coles procedure [28]. Individual epochs exceeding a maximum-minimum difference of +/−150 µV were excluded from further analysis (automatic artifact rejection as implemented in the BrainVision Analyzer software). The remaining epochs were baseline corrected with reference to a 100-ms prestimulus window, and averaged for each participant, separately for epochs with the deviant tones (averaged across the 500-Hz and 2000-Hz stimuli) and the standard tones. Trials with short and long tones were pooled, and averaged across the two test blocks to improve the signal-to-noise ratio of the EEG signal. Finally, difference waves were calculated (deviant minus standard) to analyze the deviance-related MMN, P3a, and RON components.

### Data Analysis

Behavioral and ERP data were analyzed for standard and deviant tones. RT was defined as the time between the offset of the 200-ms tone and the push of a response button. Individual RTs of less than 100 ms and more than 1200 ms, as well as error trials were excluded from further analysis. In order to define an overall performance index, a combined measurement of RT and accuracy was employed – the inverse efficiency (IE). The IE was calculated for each condition and each participant by dividing the RT by the rate of correct responses [29]. Response time and accuracy have been assumed to mirror different aspects of processing of deviancy and inhibition of distraction [30]. The IE score (expressed in ms) accounts for a possible tradeoff between speed and accuracy as it takes into account that a participant can either try to be fast, accurate, or moderately fast and accurate at the same time. Rates of correct responses, mean RTs, and mean IE of each participant were subjected to two-way analyses of variance (ANOVAs) with between-subject factor Group (active vs. inactive) and within-subject factor Stimulus (deviant vs. standard tones).

In order to examine the potential relationship between the deviant-related decline in performance and physical fitness, the correlation of the size of the participants’ individual distraction effect and power-to-weight ratio was computed. The distraction effect was defined as the percentage change in IE relative to the standard tones. It corresponds to (IEd - IEs)/IEs * 100, with IEd representing the IE to deviant tones and IEs representing the IE to standard tones. The distraction effect reflects the sensitivity to irrelevant distracting events, with high performance indicated by a small change in IE, and low performance indicated by an increase in IE to deviant tones, relative to standard tones.

The ERP analysis was restricted to midline electrodes (Fz, FCz, Cz, and Pz) chosen to be commensurate with previous knowledge of the topographical scalp distribution of specific ERPs [14], [19], indicating that the N1, MMN, P3a, RON typically peak over fronto-central areas (FCz), the P2 over central areas (Cz), and the P3b over parietal areas (Pz) of the scalp. Peak amplitudes and latencies of these components were defined as their local maximum positivity or negativity within a particular latency window (N1 at FCz: 50–150 ms; MMN at FCz: 100–200 ms; P2 at Cz: 120–220 ms; P3a at FCz: 225–400 ms; P3b at Pz: 400–700 ms; RON at FCz: 400–700 ms after tone onset). The amplitudes and latencies of these components were subjected to one-way ANOVAs with between-subject factor Group. Levene’s test was used to assess the homogeneity of variance, and the degrees of freedom were adjusted if variances were unequal. Effect sizes were computed to provide a more accurate interpretation of the practical significance of the findings, using the partial eta-squared coefficient.

### Cortical Source Localization

The neural generators of possible ERP differences between active and inactive participants were investigated using sLORETA [31]. sLORETA accounts for scalp-recorded electrical fields by dividing the brain into a three-dimensional grid of points and determining a pattern of electrical activity across these points that gives rise to the electrical fields observed at the scalp. sLORETA reduces the number of possible solutions by selecting the smoothest distribution of activity, on the assumption that the activities of neighboring brain regions are correlated. sLORETA calculates the standardized current density at each of 6239 voxels in the grey matter and the hippocampus of the Montreal Neurological Institute (MNI) brain template. The calculation is based upon a linear weighted sum of the scalp electric potentials (for details of this methodology, see [31]). sLORETA has been proven to achieve reliable localization of possible cerebral sources. The contrast of physically active and inactive participants was analyzed within a 20-ms time window around the maximum ERP peak value, using the sLORETA-built-in voxelwise randomization test (5000 permutations). This test is based on statistical non-parametric mapping (SnPM), and is corrected for multiple comparisons. Significant voxels (*p*<0.05) indicate differences between groups.

## Results

### Behavioral Data

**Figure 1:**
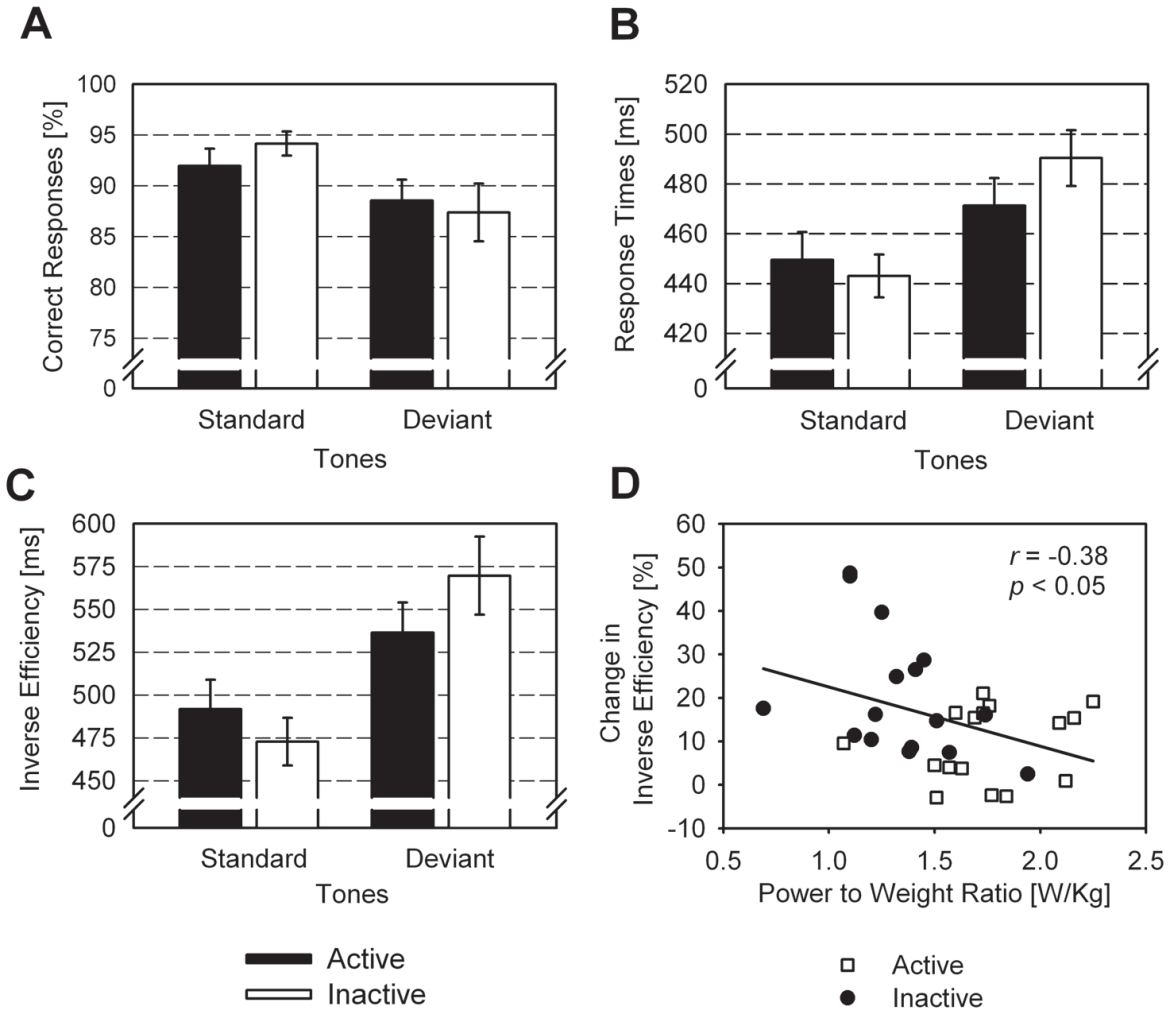
Rates of correct responses (A), response times (B), and inverse efficiency scores (C) for physically active and inactive participants, shown separately for the frequent standard tones and the rare deviant tones. Error bars indicate standard errors across participants (*N* = 16). (D) Percentage change in inverse efficiency scores (deviant tones relative to standard tones) as function of physical fitness (power to weight ratios) for each participants (N = 32), shown separately for active and inactive participants with linear regression line.

There was no main effect of Group on the percentage of correct responses (*F* [1], [30] = 0.46; *p*>0.05; η_p_^2^ = 0.01) and no interaction of Stimulus and Group (*F* [1], [30] = 1.62; *p*>0.05; η_p_^2^ = 0.05), while a main effect of Stimulus (*F* [1], [30] = 14.98; *p*<0.001; η_p_^2^ = 0.33) indicated a higher rate of correct responses with standard, than deviant, tones (Fig. 1A). There was no main effect of Group on RTs either (*F* [1], [30] = 0.20; *p*>0.05; η_p_^2^ = 0.01), but a main effect of Stimulus (*F* [1], [30] = 61.28; *p*<0.001; η_p_^2^ = 0.67) indicated higher RTs with deviant, than standard, tones. Moreover, there was a significant interaction of Group and Stimulus (*F* [1], [30] = 8.38; *p*<0.01; η_p_^ 2^ = 0.22), resulting from inactive participants showing a more pronounced increase in RTs with the deviant tones than active participants (Fig. 1B). Regarding the overall IE score, there was no main effect of Group (*F* [1], [30] = 0.01; *p*>0.05; η_p_^2^ = 0.01), but a significant Group x Stimulus interaction (*F* [1], [30] = 6.96; *p*<0.05; η_p_^2^ = 0.19) and a main effect of Stimulus (*F* [1], [30] = 50.07; *p*<0.001; η_p_^2^ = 0.63). Thus, while the deviant tones impaired performance of both groups, relative to standard tones, inactive participants showed a stronger decline than their active counterparts (Fig. 1C). Accordingly, there was a significant between-group difference in the percentage changes in IE scores (active: 9.5%, SE 2.1%; inactive: 20.6%, SE 3.6%; *F* [1], [30] = 7.05; *p*<0.05; η_p_^2^ = 0.19). Moreover, there was a significant correlation of the participants’ power-to-weight ratios and the percentage changes in IE scores (*r* = −0.38; *p*<0.05), suggesting a relation between physical fitness and the deviance-related decline in performance (Fig. 1D).

### ERPs

**Figure 2:**
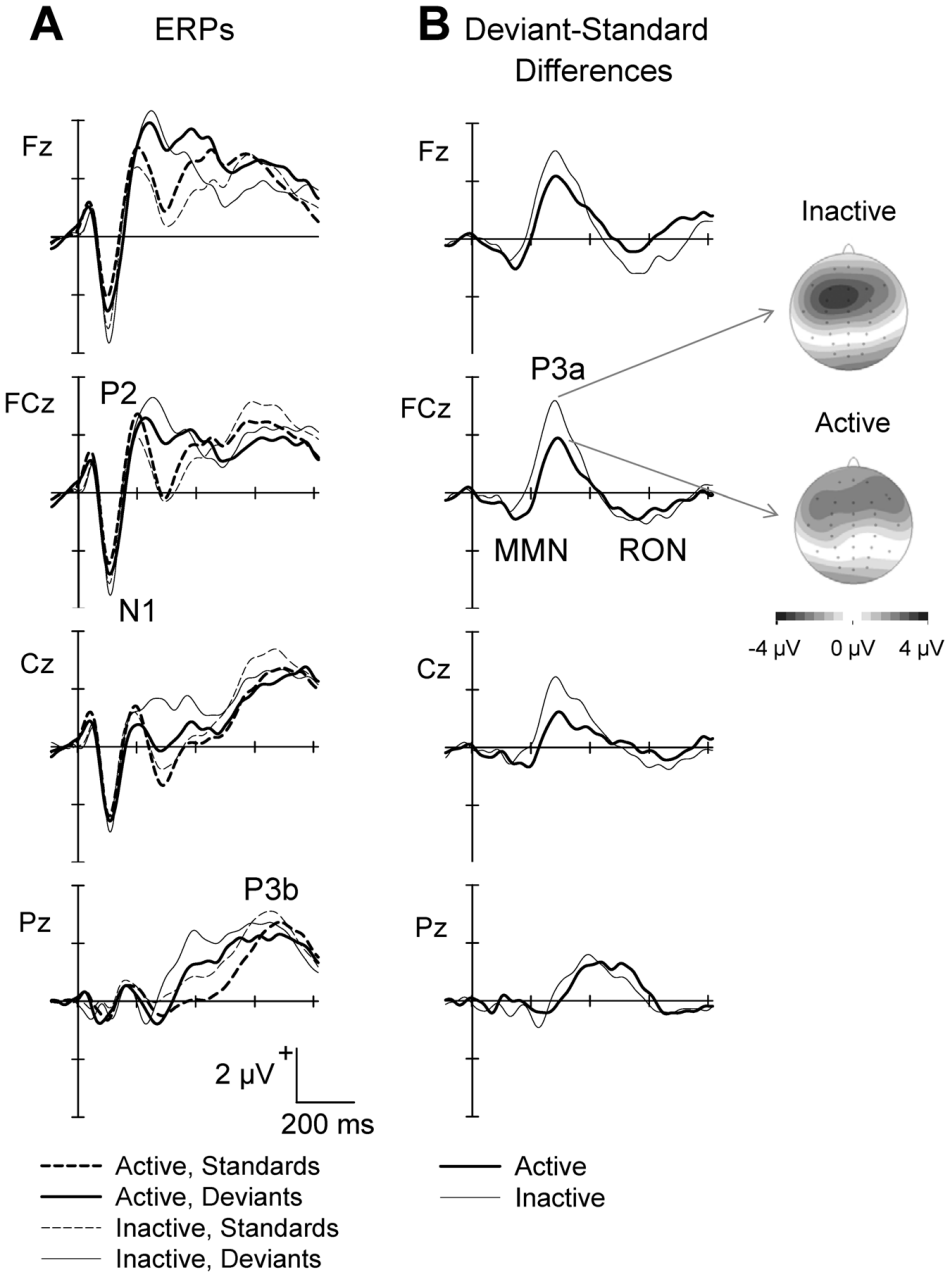
Grand average ERPs of the frequent standard tones and the rare deviant tones (A), and difference waves (deviant minus standard tones; B) at Fz, FCz, Cz, and Pz, shown for physically active and inactive participants. The vertical lines reflect the onset of the tone stimulus. ERP components (N1, P2, P3a, P3b, MMN, and RON) are marked at the waveform of maximal amplitude. The topographies represent the P3a for active (bottom) and inactive (top) participants.

Grand average ERP-waveforms for standard and deviant tones at Fz, FCz, Cz, and Pz are shown in Figure 2A for each group. Standard tones produced a typical fronto-central N1-P2 complex peaking at 93 ms and 185 ms, respectively, and a pronounced parietal P3b peaking at 633 ms. Deviant tones produced, beside a fronto-central N1 and a parietal P3b, a strong fronto-central positivity at about 300 ms after tone onset. The difference waveforms (deviant minus standard tones) shown in Figure 2B indicated a fronto-central MMN (peaking at 145 ms), P3a (peaking at 294 ms), and RON (peaking at 562 ms after tone onset).

The analysis of the ERP to the standard did not indicate any significant between-group difference in N1, P2, or P3b amplitudes or latencies (Tab. 2). Furthermore, there were no significant N1 differences in response to the deviant tones. However, the analysis of the difference waveforms indicated a significantly stronger P3a in inactive participants, relative to active participants. No significant differences occurred in MMN or RON amplitudes or latencies. Thus, while active and inactive participants did not differ in the processing of standard tones, the deviance-related fronto-central positivity P3a was more pronounced in the inactive than active group of participants.

**Table 2:** Mean amplitudes (Amp. in µV) and latencies (Lat. in ms) and standard error of means of the event-related potentials of physically active and inactive participants for standard and deviant tones, with *F* statistics and effect sizes.

		Active	Inactive	*F* (1,30)	*η_p_^2^*
Standard Tones	N1 Ampl.	−3.51 (0.51)	−4.18 (0.70)	0.59	0.02
	N1 Lat.	91.9 (2.9)	93.5 (1.9)	0.22	0.01
	P2 Ampl.	3.23 (0.48)	2.52 (0.64)	0.78	0.03
	P2 Lat.	186.2 (3.4)	183.7 (4.4)	0.19	0.01
	P3b Ampl.	3.17 (0.42)	3.67 (0.52)	0.47	0.02
	P3b Lat.	638.7 (9.8)	627.2 (10.8)	0.62	0.02
Deviant Tones	N1 Ampl.	−4.17 (0.41)	−4.58 (0.70)	0.26	0.01
	N1 Lat.	97.5 (4.7)	95.5 (1.79)	0.16	0.01
	MMN Ampl.	−2.01 (0.33)	−1.51 (0.32)	1.18	0.04
	MMN Lat.	150.8 (5.9)	138.2 (5.2)	2.57	0.08
	P3a Ampl.	2.93 (0.32)	4.86 (0.48)	11.11**	0.27
	P3a Lat.	292.6 (6.8)	295.1 (9.1)	0.05	0.01
	RON Ampl.	−2.43 (0.31)	−3.09 (0.37)	1.85	0.06
	RON Lat.	559.9 (14.0)	563.9 (18.1)	0.03	0.01

MMN, P3a, and RON refer to the difference event-related potentials (deviant minus standard tones).

** *p*<0.01.

### Full-brain Source Analysis of P3a

**Figure 3:**
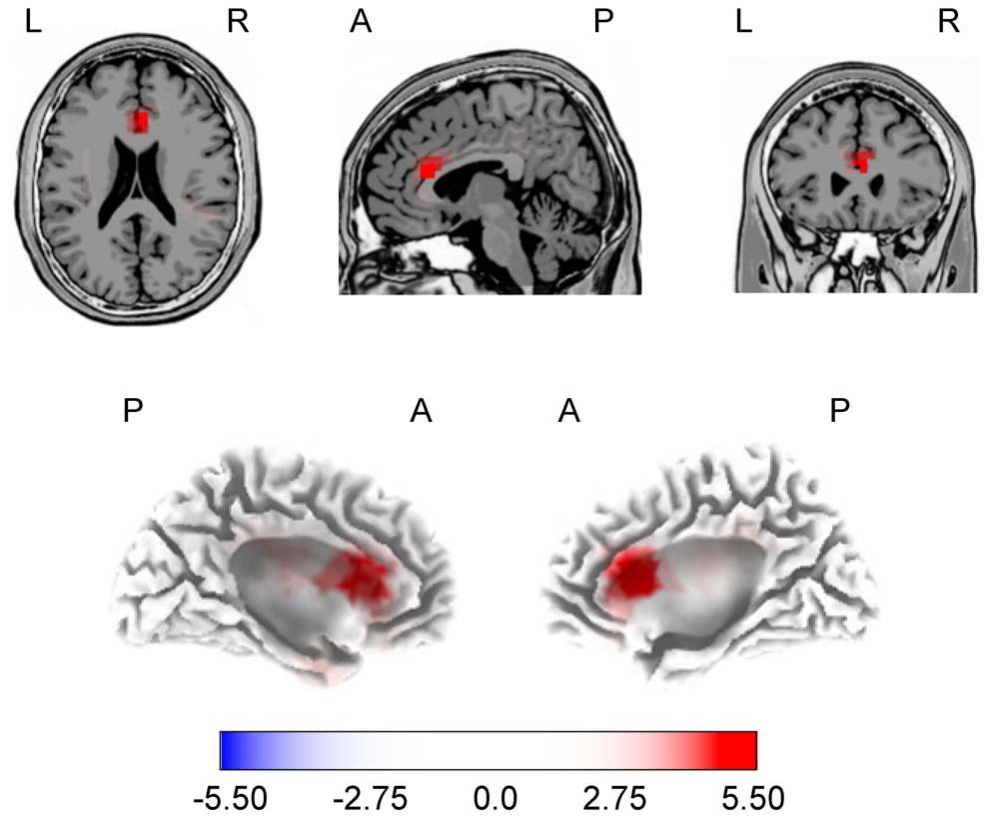
Activation of brain regions as revealed by sLORETA analysis. P3a responses of the physically active and inactive groups were contrasted and tested for significance using the sLORETA-built-in voxelwise randomization test. The statistical cut-off was set to a significance level of *p* = 0.05. Data from all participants were projected onto a single anatomical image (MNI-template of sLORETA) with transversal, sagital, and coronal slices positioned at Talairach xyz-coordinates of 5, 25, 20 mm, respectively, to focus on maximum activation (upper row) and 3D cortical surface (lower row). A anterior, P posterior, L left, R right.

In order to reveal the cortical sources of the between-group difference of P3a, the difference waveforms of the active and inactive groups were contrasted within a 20-ms window around the participants’ individual P3a peak latencies. The contrast indicated significant activation mainly in right Anterior Cingulate. Significant, but less extensive, activation was also found in left Precentral Gyrus and left Anterior Cingulate (Fig. 3; Tab. 3). The contrast indicated stronger ACC activation in inactive, than active, participants, whereas no single voxel indicated stronger activation of active, than inactive, participants. Thus, the more pronounced P3a in the inactive participants was mainly associated with a stronger involvement of Anterior Cingulate.

**Table 3:** Locations of significant activation evoked by deviant stimuli relative to standard stimuli for the P3a deflection.

	Talairach coordinates [mm]			BAs	*t*-value	number of voxels
Region	x	y	z			
Right Anterior Cingulate	5	25	17	24, 32, 33	5.56	14
Left Precentral Gyrus	−35	−12	42	6, 4	4.79	3
Left Anterior Cingulate	−5	21	22	24	4.51	2

Activations resulted from contrast of physically active and inactive participants, as revealed by sLORETA. Coordinates are in standard stereotaxic space [61] and refer to maximally activated foci, as indicated by the highest t-score within an area of activation. Number of voxels refers to significant voxel (*t*-value >4.48; *p*<0.05). BAs, Brodmann Areas.

### Correlation Analysis

**Figure 4:**
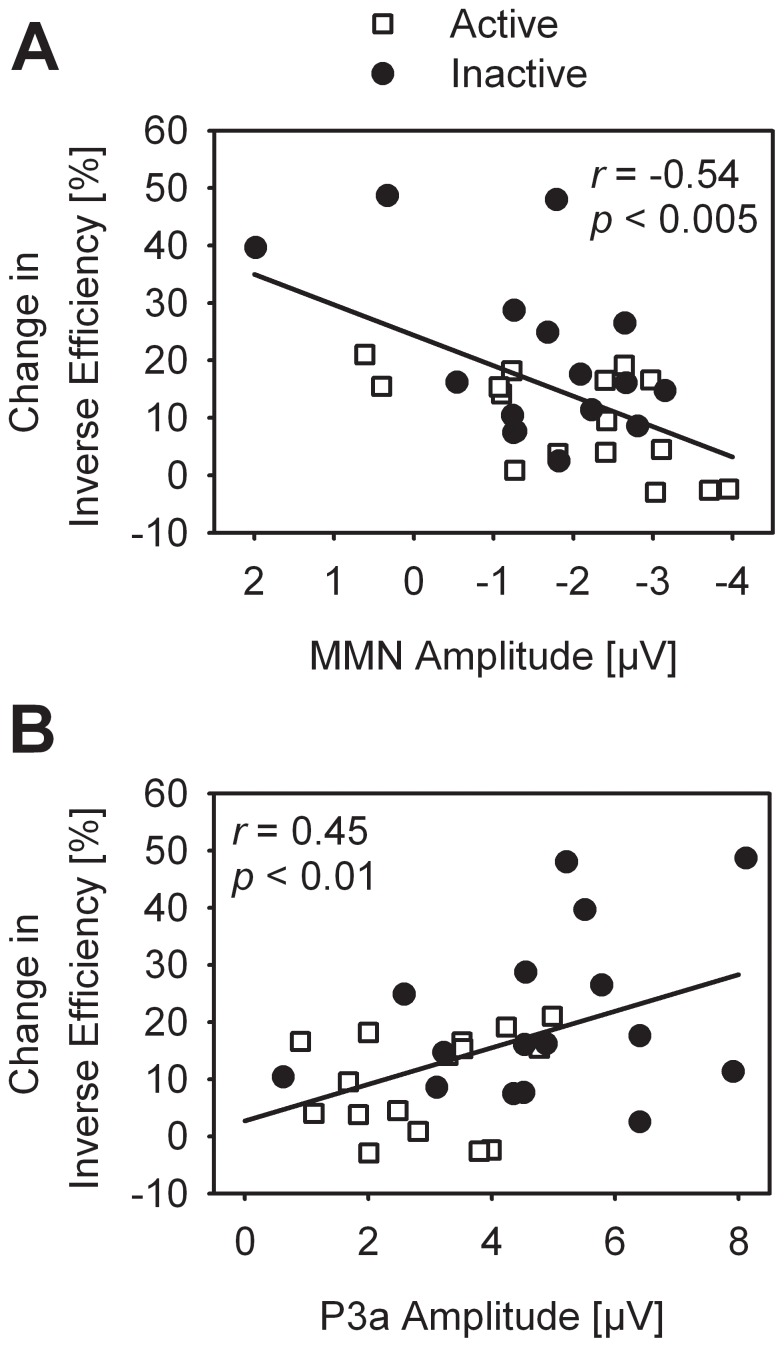
Percentage change in inverse efficiency scores (deviant tones relative to standard tones) as function of MMN amplitudes (A) and P3a amplitudes (B) at FCz for each participant (N = 32), shown separately for physically active and inactive participants with linear regression lines.

To investigate a potential relationship between the deviance-related decline in performance and ERPs, correlations of the size of the participants’ individual distraction effect and MMN, P3a, and RON amplitudes and latencies were computed. There were significant correlations of change in IE and MMN amplitude (*r* = −0.54; *p*<0.01; Fig. 4A), and of change in IE and P3a amplitude (*r* = 0.46; *p*<0.05; Fig. 4B). The percentage change in IE was not correlated with RON amplitude (*r* = 0.07; *p*>0.05), nor with ERPs latencies (all *p*>0.05; all values Bonferroni-Holmes corrected). Thus, the deviance-related decline in performance in duration discrimination was associated with a stronger P3a and a weaker MMN amplitude.

## Discussion

Previous studies reported beneficial effects of physical activity and aerobic fitness on brain and cognition, with the strongest and most consistent effects on executive functions [32], [33]. Here, an association of physical fitness and auditory distraction in seniors was shown in a duration discrimination task. While physically active and inactive participants did not differ in performance per se (i.e., in response to standard or deviant stimuli) the decrease in performance in response to deviant stimuli, relative to standard stimuli, was more pronounced in inactive participants. Moreover, a significant correlation of deviance-related change in inverse efficiency and performance in ergometry indicated that the strength of distraction was related to the participants’ individual physical fitness. Thus, the present results suggest a positive relationship between physical fitness and orientation-related attentional processes in seniors. In order to clarify which cognitive sub-processes within the distraction-orientation-refocusing cycle contributed most to the performance difference, the ERPs of active and inactive seniors were analyzed.

### Increased Deviance-related P3a in Inactive Seniors

The contrast of standard and deviant related processes indicated a more pronounced frontal P3a in inactive than active seniors, suggesting that the cognitive sub-process reflected by the P3a differed between the two groups. The P3a is assumed to be a correlate of selection of stimulus information governed by attentional orienting, indicating the disengagement of previous attentional focus to re-engage attentional processes towards an infrequent deviant stimulus [34], [35]. Besides an involuntary (automatic) shifting, some recent studies have suggested that the P3a could also reflect voluntary attentional orientation [19], [36]. Within the framework of the three-stage model of distraction, the P3a is a correlate of the attention-switching mechanism, with larger amplitudes associated with greater attentional orienting towards the deviant stimulus feature and – consequently – more distraction from the ongoing task [12], [15], [16]. Thus, it appears that the deviant stimulus feature distracted inactive participants more than active participants in a way that inactive participants processed the task-irrelevant deviant feature more intensely than their active counterparts. This could consume more attentional resources, leaving fewer resources to adequately perform the main task. This assumption is supported by a positive correlation of P3a amplitude and change in IE (cf. Fig. 4B), indicating that a strong distraction effect was associated with a pronounced P3a amplitude.

There were no group differences in MMN amplitude or latency. The MMN is regarded as a correlate of early processes of standard formation and detection of changes in the acoustic environment that may induce a listener’s orientation to potentially relevant information by capturing his or her attention (for review: [14], [37]). Previous studies revealed a decrease in MMN in aging [20], [21], [38], suggesting these processes to be less efficient in seniors. Deficient standard formation and deviance detection may in turn lead to a more controlled processing of changes in the auditory environment. In line with this notion, it has been suggested that the decrease in MMN in elderly could reflect a switch from automatic to controlled processing of sound in aging, in a way that declines in automatic detection of small change in the environment could be compensated for by top-down controlled processes [39]. In the present distraction task, more controlled processing of the task-irrelevant deviant sound feature (P3a) could be related to a more severe performance decline in the responses to the relevant sound feature of the deviant stimuli. In line with this notion, a strong change in IE was associated with a weak MMN amplitude (cf. Fig. 4A). In turn, participants with a pronounced MMN showed only a mild deviance-related decline in performance. This relationship between MMN and size of behavioral distraction did not depend on physical fitness, however. Taken together, it appears that both the MMN (reflecting early standard formation and deviance detection) and the P3a (reflecting attention-switching toward the deviant information) were intimately related to the deviance-related decline in performance, while only the later process in the distraction-orientation-refocusing cycle was associated with physical fitness.

There was no relation of behavioral distraction and physical fitness to the amplitude or latency of the RON. Within the framework of the three-stage model of distraction, the RON is assumed to reflect a re-allocation mechanism of attention [10]. Age-related decreases in RON amplitude and increases in RON latency has thus been interpreted as evidence that the triggering of the attention-switching mechanism is less efficient and takes longer in elderly [6], [7]. Given that there were no significant RON differences between active and inactive participants, the re-orientation process was obviously not related to the participants’ physical fitness. The present pattern of results indicated an obvious dissociation of MMN, P3a, and RON, suggesting that the processes reflected by these ERPs are not strongly coupled. The presence of an association between P3a, physical fitness, and auditory distraction in the absence of MMN and RON effects rather suggests that the cognitive sub-processes of the distraction-orientation-refocusing cycle can at least partly run independently. This notion is corroborated by previous evidence that also argue against a strongly coupled chain of auditory distraction and involuntary control of attention (e.g., [40], [41]).

Taken together, the present results corroborate the findings of a recent study in which electrophysiological correlates of individual differences in auditory distraction were investigated in younger and older adults [22]. Low-performing seniors (i.e., participants with the strongest deviance-related decline in performance) showed a larger P3a amplitude than high-performing seniors (participants with only mild deviance-related decline in performance), whereas MMN and RON did not differ between the two older groups. Thus, high- and low-performing seniors showed a similar pattern of ERP results as the physically active and inactive participants in the present study.

It should be noted that the present design was cross-sectional. Although physically active and inactive participants were carefully matched on age, educational level, and sex, it cannot fully be excluded that further variables contributed to the differences found in performance and ERPs. However, the analysis of a number of physical and neuropsychological variables did not indicate significant differences between the two groups. In addition, the significant correlation between the participants’ fitness scores and the individual amount of distraction argued for a direct relationship between physical fitness and the cognitive variables. Nevertheless, longitudinal training studies with random assignment are needed to further corroborate the supposed beneficial effect of physical exercise on auditory attentional control in elderly.

### ERPs to Standard and Deviant Tones

No significant differences were found in N1 and P2 amplitudes or latencies, neither in response to standard, nor deviant tones. The N1-P2 complex has multiple generators in the supra-temporal plane, in or near primary auditory cortex (e.g., [17], [42]). While the N1 reflects early processes of stimulus detection in the acoustic environment [17], the role of P2 is less clear. It has been related to processes of stimulus evaluation and early attention allocation [18]. However, the P2 has also been shown to be modulated by sensory features, e.g. by spectral complexity and music training [43], by laboratory based discrimination training [44], [45], or by frequency separation of tones in a stream segregation task [46]–[48]. Given that there were no significant differences between active and inactive participants, it appears that these early processes of stimulus detection and evaluation were not directly related to the participants’ physical fitness.

Interestingly, there was also no difference in the parietal P3b. The P3b is assumed to be a correlate of allocation of working memory and processing resources [19]. Previous findings of age-related increases in P3b latency and decreases in P3b amplitudes have thus been interpreted as evidence of a general cognitive slowing [49] and reduced allocation of processing resources in elderly [19], [49], [50]. However, active and inactive seniors in the present study did not appear to differ in speed of cognitive processes or in processing capacity. Moreover, fitness-related differences in cognitive slowing and processing capacity should also be reflected in behavioral performance, but there were no group differences in RT or accuracy. These findings are in contrast to results of previous studies, which found shorter P3 latencies or higher P3 amplitudes in physically active than inactive seniors [5], [51]. On the other hand, Pontifex et al. also found no P3 latency effect between active and inactive seniors [35], supporting our findings. This discrepancy may be due to differences in the experimental setting. For example, the difficulty of the background task applied in the present study was relatively low, and fitness effects are known to be the maximum for difficult tasks including executive functions [4]. Another reason for the lack of P3b effects in the present study may be the use of auditory modality, since as far as we know in all previous studies visual stimuli were used. Auditory duration discrimination may be easier than visual discrimination, which may have attenuated the group differences. In line with this notion, a previous ERP study in which an auditory and visual oddball task was employed to examine the association between physical activity level and cognitive function in young adults found much stronger effects of physical exercise on P3 amplitude in the visual than auditory modality [52]. To further test these hypotheses an auditory and a visual version of our task with carefully matched task difficulty across modalities could be employed in a future study.

### The Role of the ACC

Cortical source localization revealed that the fitness-related difference in P3a amplitude was associated with ACC activation, being stronger in inactive than active participants. While the ACC has classically been related to affect, neuroimaging research revealed two functionally distinct subdivisions of ACC, a rostral-ventral affective division and a dorsal cognitive division [53]. The latter one has been related to various cognitive functions, including modulation of attention and executive functions, particularly novelty and error detection, conflict resolution, response selection, and working memory [53]–[55]. In particular, increased activation of the ACC was associated with task difficulty [56], and was found with tasks in which subjects had to pay attention to an unexpected or novel stimulus, or in which subject had selectively attend to one stimulus, while inhibiting the response to another stimulus [15]. Moreover, the ACC has been found to be involved in generation of the P3a [19], [57], [58]. Taken together, the ACC appears to be part of a cortical network that – besides emotional regulation – is involved in regulation of cognitive processes that play an important role in attentional control [53], [57]. In combination with the stronger behavioral distraction effect of the inactive than active seniors in the present study, the difference in ACC activation suggested an adverse allocation of attentional resources towards irrelevant stimulus features.

This notion is in line with results of an fMRI study, in which a flanker task was employed that required executive control to inhibit the distracter stimuli (flanker) and to enhance the attentional processing of the target stimuli. A decrease in ACC activation was found in aerobically trained older adults, relative to untrained participants, that was associated with a more efficient performance in dealing with conflicting stimuli [59]. While this reduction in ACC activation has been associated with reduced conflict processing, the results of the present auditory distraction task rather suggested enhanced attentional control of the physically active participants. The present results are also in line with a recent ERP study indicating beneficial effects of physical activity on behavioral and neuroelectric indices of action monitoring and executive control [60].

### Conclusion

There was a positive relationship between physical fitness and specific cognitive sub-processes involved in the distraction-orientation-refocusing cycle that are necessary to establish smoothly unfolding of behavioral control. Relative to physically active seniors, inactive seniors showed an increased allocation of attentional resources to the irrelevant aspects of deviant stimuli, which could distract resources from processing of, and response to, the relevant aspect. This indicated a lower auditory distractibility of physically active compared to inactive seniors. Further studies – including visual as well as auditory stimuli – should investigate whether this difference in distractibility is modality-specific or not.
